# Multi-physical field simulation calculation and analysis of simulated high-level waste liquid spray calcination

**DOI:** 10.1371/journal.pone.0308145

**Published:** 2024-08-09

**Authors:** Yanmei CHENG, Kening LI, Xingyun JIA, Guoan YE

**Affiliations:** 1 China Institute of Atomic Energy, Beijing, China; 2 Ministry of Chemical Safety Education Engineering Research Centre, Beijing University of Chemical Technology, Beijing, China; Federal University of ABC, BRAZIL

## Abstract

Aiming at the independent research and development of a simulated high-level waste liquid spray calcination transformation treatment test device, a three-dimensional multi-physical field model of spray calcination was established by means of finite element analysis method. In this paper, the simulated high-level waste liquid is a mixed solution of nitrate solution and sucrose. The main chemical components of nitrate dissolution are HNO_3_ and NaNO_3_. The process of evaporation and calcination of high-level waste liquid to form oxides is also called the pretreatment of high-level waste liquid or the conversion of high-level waste liquid. In this experiment, the atomized droplets sprayed at high speed are evaporated, dried and calcined in turn in the calciner to obtain the calcined product. The distribution law of temperature flow field and chemical reaction state and results inside the test device were revealed by simulation calculation. The results show that under the condition of multi-physical field coupling, the chemical reaction temperature has an effect on the yield of the product. The temperature is positively correlated with the product concentration, and the effect of temperature on the yield of NO_2_ is greater than that of Na_2_O. At the same time, in this chemical reaction, the concentration of reactants (NaNO_3_ and HNO_3_) had a positive correlation with the concentration of main products (NO_2_ and Na_2_O). However, the rate of increase in the concentration of the main products (NO_2_ and Na_2_O) decreased with the increase of the concentration of the reactants (NaNO_3_ and HNO_3_).

## 1. Introduction

The high-level waste liquid produced after spent fuel treatment [1, 2] still contains more than 99% of fission products and minor actinides in spent fuel [3]. High-level liquid waste has the characteristics of complex composition, strong radioactivity, strong corrosion and strong toxicity [4], and its treatment and disposal has always been one of the key and difficult problems in the nuclear industry. At present, the widely recognized and relatively mature technology in the world is the glass solidification technology of high-level liquid waste [3, 5–7]. China Institute of Atomic Energy has made relevant research and development on the two-step cold crucible glass curing technology [8, 9]. The transformation technology of high-level waste liquid described in this paper is the first step of the two-step cold crucible glass curing technology. Specifically, it refers to the evaporation and calcination of high-level waste liquid under high temperature conditions, and finally the formation of solid calcinations products [10].

At present, there are many methods of independent pretreatment of high-level liquid waste calcination process, such as tank calcination method, rotary furnace calcination method, fluidized bed calcination method, microwave calcination method and spray calcination method [11]. The spray calcination equipment has the advantages of simple structure, convenient operation and maintenance, and high calcination efficiency (the high-level waste liquid can be directly dried into powder, and the drying process is rapid). Based on the spray calcination method, American researchers have developed a spray-type high-level waste liquid transformation experimental device for waste liquid transformation treatment [12]. Under the background of the international verification of this process flow, based on the spray calcination method, this paper independently developed a simulated high-level liquid waste spray calcination transformation treatment test device for simulated high-level liquid waste transformation treatment experimental research, which provides a good research platform for this treatment technology.

Over the years, domestic and foreign scholars have carried out a large number of experiments and numerical simulations on spray drying technology. Bass Ler [13] proposed the spray drying method to prepare milk powder. Wu and Liu [14] used the strong oscillating flow field generated by the pulse combustor to spray dry the sodium chloride solution, and used the computational fluid dynamics method to simulate the momentum, heat and mass transfer processes of the gas phase and the particle phase, and discussed the influence of the airflow pulsation frequency on the drying process. Oluwafemi et al. [15] deeply explored the local characteristics of evaporation rate at the interface between droplet and air by means of single droplet drying (SDD), and successfully obtained the distribution information of temperature and evaporation rate inside droplet. Roman et al. [16] deeply analyzed the correlation between the evaporation rate of atomized droplets and the temperature and velocity of airflow through fluorescence optical diagnostic technology. In addition, numerical simulation software (CFD) has shown great ability in describing the evaporation process of droplet group, which has been applied to the study of wastewater evaporation by researchers, and remarkable progress has been made [17, 18]. The problem of traditional simulation is that the heat transfer calculation is calculated for a single flow field, and the coupling of chemical reaction is not considered. Therefore, this paper considers the coupling effect of flow heat transfer and chemical reaction simulation.

## 2. Numerical calculation method and calculation model

### 2. 1 Flow field model

The ’turbulence, k-ε’ model is suitable for fully turbulent, medium to high pressure range and high viscosity fluid models. This model is a turbulence model. The ’turbulence, k-ε’ model is widely used in industry because of its wide applicability, economy and reasonable accuracy. For most applications, the k-ε model can provide stable and reliable simulation results. In engineering calculation, the calculation amount of this model is moderate, and the calculation accuracy and convergence are reasonable. After the above analysis, the ’turbulence, k-ε’ model is selected.

The ’turbulent, k-ε’ model is used to simulate the fluid motion inside the test device. In addition, considering that the flow rate of the liquid inside the test device is relatively low and the pressure change is relatively small, it is regarded as an incompressible Newtonian fluid. Because the overall fluid flow process inside the device is the flow provided by the negative pressure at the outlet, the influence of gravity can be ignored. The mathematical model is described as follows:

ρ(u⋅∇)u=∇⋅[−pI+K]+F (1)

ρ∇⋅u=0 (2)

$$\text{K}=\left(\mu +\mu_{\text{T}}\right)\left[\nabla \text{u}+{\left(\nabla \text{u}\right)}^{\text{T}}\right]$$ (3)

$$\rho (\text{u}\cdot \nabla )\text{k}=\nabla \left[\left(\mu +\frac{\mu_{\text{T}}}{\sigma_{\text{k}}}\right)\nabla \text{k}\right]+{\text{P}}_{\text{k}}-\rho \varepsilon$$ (4)

$$\rho (\text{u}\cdot \nabla )\epsilon =\nabla \left[\left(\mu +\frac{\mu_{\text{T}}}{\sigma_{\epsilon }}\right)\nabla \epsilon \right]+{\text{C}}_{\epsilon 1}\frac{\epsilon }{\text{k}}{\text{P}}_{\text{k}}-{\text{C}}_{\epsilon 2}\rho \frac{\epsilon^{2}}{\text{k}},\epsilon =\text{ep}$$ (5)

$$\mu_{T}=\rho C_{\mu }\frac{k^{2}}{\epsilon \epsilon_{\text{p}}}$$ (6)

$${\text{P}}_{\text{k}}=\mu_{\text{T}}\left[\nabla \text{u}:\left(\nabla \text{u}+{\left(\nabla \text{u}\right)}^{\text{T}}\right)\right]$$ (7)

In the formula: *u* is the fluid velocity; *p* is the fluid pressure, Pa; *I*is the unit matrix; *K* is the expansion of stress term; *F* is the volume force vector; *k* is turbulent kinetic energy; *ε* is the turbulent dissipation rate; *μ*_T_ is turbulent viscosity; *P*_k_ is the expansion of the generating term; *σ*_k_, *σ*_ε_, *C*_μ_, *C*_ε1_ and *C*_ε2_ are all empirical constants. *▽* is Hamiltonian operator; *u* = (*u*_x_, *u*_y_, *u*_z_), *u*_x_, *u*_y_, *u*_z_ are the *x*, *y*, *z* axis components of the three-dimensional space vector *u*.

### 2. 2 Thermal model

The ’solid + fluid heat transfer model’ is used to simulate heat transfer through conduction, convection and radiation in solids and fluids. During the spray calcination process, the heat exchange between the droplet (fluid) and the hot surface (solid) involves two main mechanisms: heat conduction and heat convection. This is consistent with the heat transfer mechanism described by the solid + fluid heat transfer model. Thermal conduction occurs inside the solid or between solids, while thermal convection occurs between the solid surface and the contact fluid. Therefore, the ’solid + fluid heat transfer model’ is adopted.

The wall of the calciner body of the equipment provides a high-temperature heat source. There is heat exchange between the calciner, the product hopper and the filter, and there is heat exchange between the injected feed liquid and the test device. Because there are both solid and fluid in the whole model domain, the ’solid + fluid heat transfer’ model is used to simulate the heat transfer process. The transient mathematical model is described as:

$$\rho {\text{C}}_{\text{p}}\text{u}\cdot \nabla \text{T}+\nabla \cdot \text{q}=\text{Q}+{\text{Q}}_{\text{ted}}$$ (8)

q=−k∇T (9)

In the formula: *C*p is the constant pressure heat capacity, J/(kg·K); *q* is the heat flux, W/m^2^; *k* is the thermal conductivity, W/(m·K); *Q* is the heat source term; *Q*_ted_ is thermoelastic damping; *T* is temperature, K.

### 2. 3 Reaction model

The transport of dilute substances (tds) is used to calculate the concentration field of dilute solutes in solvents. The transport and reaction of substances dissolved in gas, liquid or solid can be processed using this interface. The spray calcination model in this paper can simulate the concentration of reactants and products at different temperatures through the dilute material transfer module.

The transfer and reaction in the reaction channel are described by the mass conservation given by the steady-state convection-diffusion equation:

$$\nabla \cdot \left(-{\text{D}}_{\text{i}}\nabla {\text{c}}_{\text{i}}\right)+\text{u}\cdot \nabla {\text{c}}_{\text{i}}={\text{R}}_{\text{i}}$$ (10)

In the formula: *D*_i_ denotes the diffusion coefficient, m^2^/s; *c*_i_ is the substance concentration, mol/m^3^; *u* is the velocity vector, m/s. *R*_i_ (mol/(m^3^·s)) is equivalent to the reaction rate expression of the substance.

## 3. Multi-physical field coupling calculation model of spray calcination

### 3. 1 Three-dimensional model

**Fig 1:**
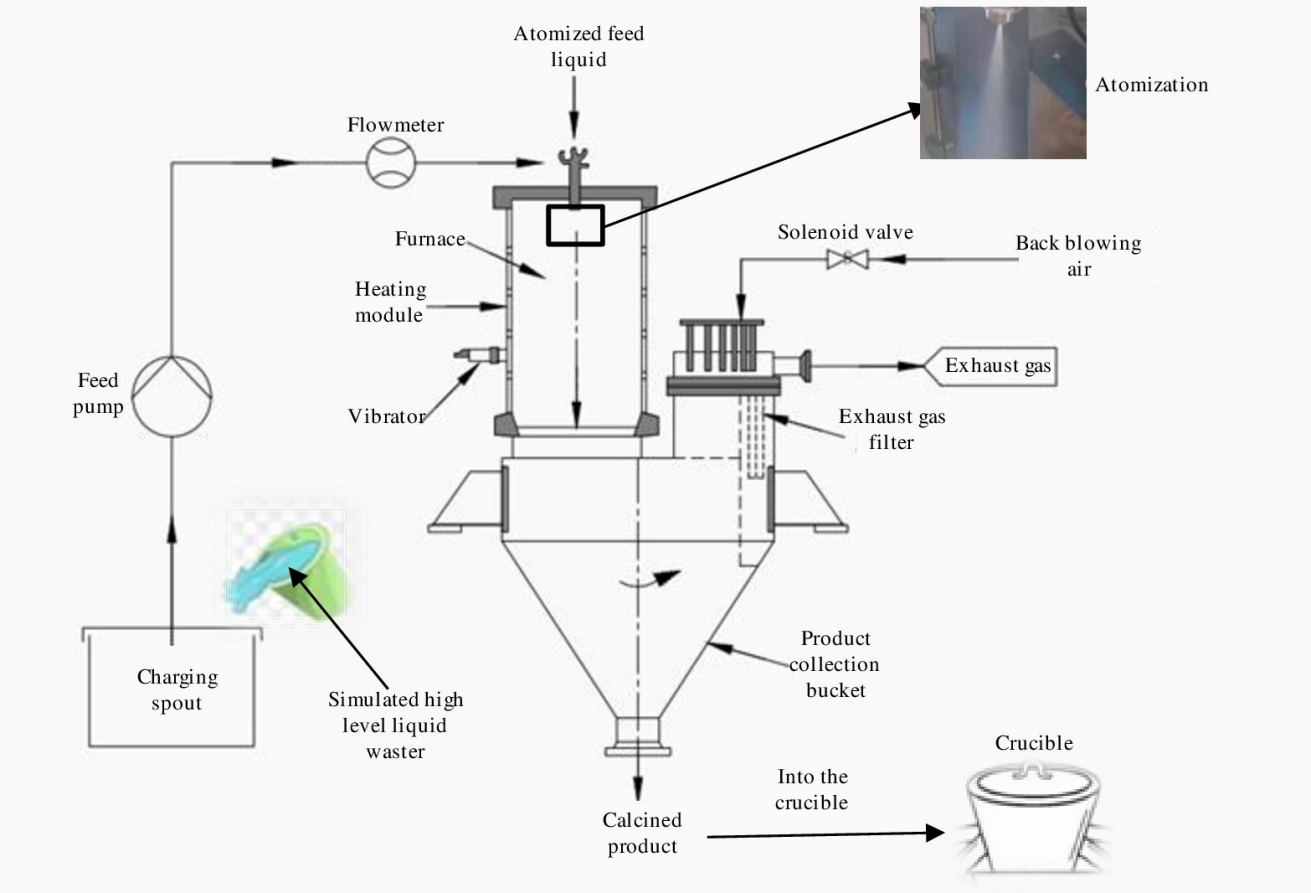
Process flow diagram of simulated high-level waste liquid spray calcination transformation treatment test device.

The model described in this paper is a high-level waste liquid spray calcination transformation treatment test device, which is mainly composed of a feeding system, a spray calcination system, an exhaust gas filtration system and a product collection system. The process diagram is shown in Fig 1. Firstly, the waste liquid is sprayed into the furnace chamber from the top of the calcination chamber in the form of droplets by the atomization device. The droplets are evaporated, dried and calcined vertically through the calcination chamber. At the same time, the intermittent vibration of the vibrator installed on the outside of the furnace can reduce the deposition and scaling of the material on the surface of the furnace. The calcined product falls into the hopper for storage or direct glass curing. The steam produced in the calcination process is filtered by the tail gas filter. The filtered tail gas enters the tail gas purification treatment system for washing, absorption and other operations, and finally discharges to the atmosphere after meeting the emission standards. In order to stop the filter blockage and reduce the filtration pressure difference, the tail gas filter needs to have a hot air reverse blowing function to blow the calcined product attached to the filter back to the product receiving hopper.

**Fig 2:**
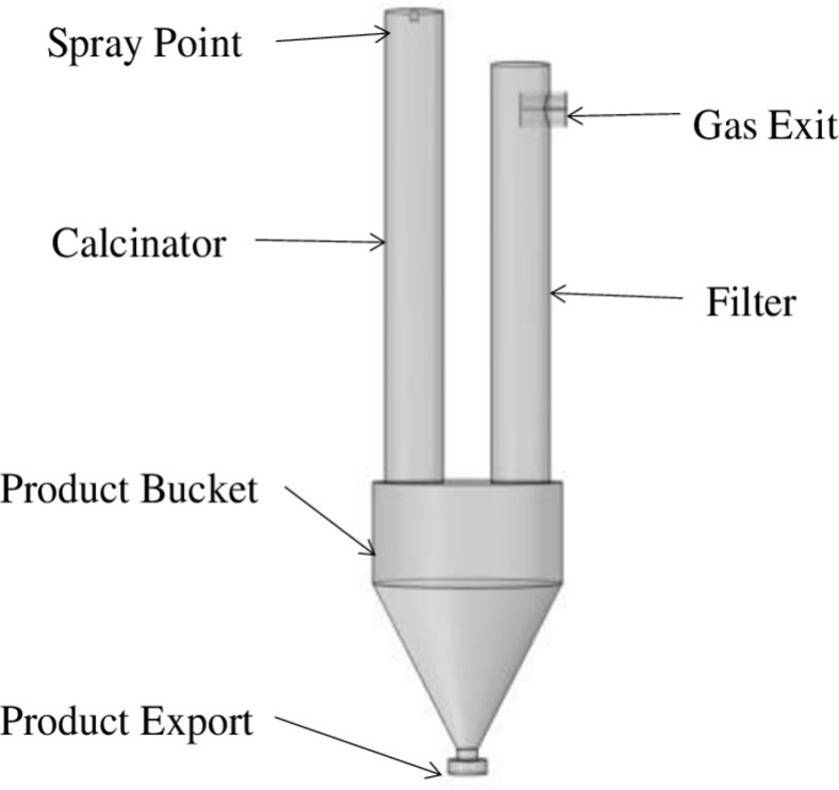
Simulation geometric model of spray calcination experimental device.

**Fig 3:**
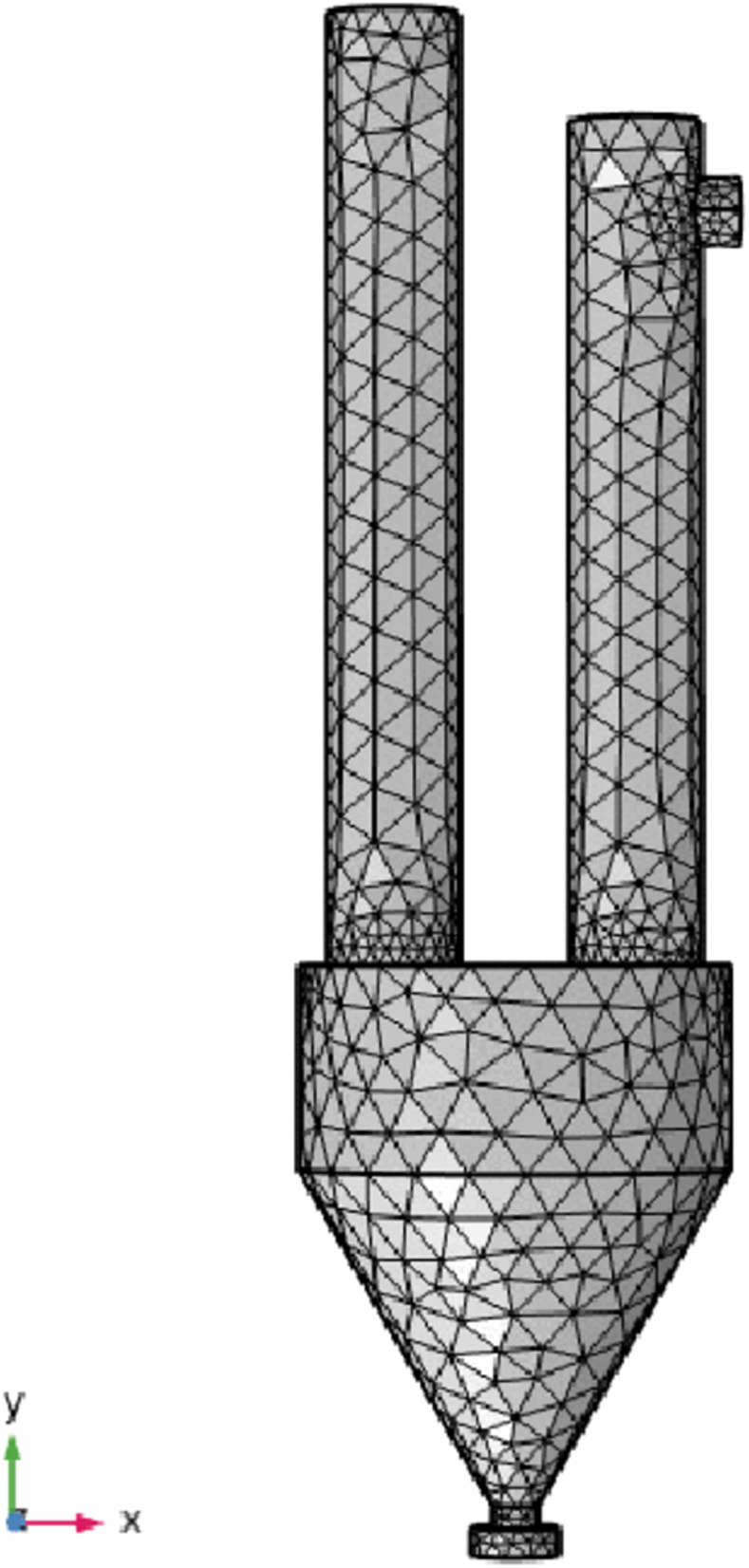
Grid subdivision diagram of geometric model.

The simulation object of this part is the main part of the spray calciner. According to the design size of the industrial model, a multi-physical field coupling calculation area including the spray calcination system, the product hopper and the exhaust gas filtration system is established, as shown in Fig 2. The tetrahedral element is used to mesh the geometric model of the whole fluid calculation domain, and the grid independence analysis is carried out. The total number of grid vertices is 7407, the number of grids is 21050, and the average unit mass is 0.6244. The grid quality is good, and the grid division of the multi-physical field coupling calculation area is shown in Fig 3.

### 3. 2 Boundary condition setting

In the simulation, the ’turbulent, k-ε’ model is used to simulate the internal flow field, and the spray point is used as the ’inlet’ boundary condition. The inlet normal inflow velocity is 3m/s, the turbulence intensity is specified as medium (0.5), and there is no slip on the inner wall. The filter outlet is used as the ’outlet’ boundary condition. The filter outlet is provided with negative pressure by the fan during the actual operation, and the boundary condition of the outlet is negative pressure -2000 Pa.

In the simulation, the ’solid + fluid heat transfer’ model is used to simulate the internal thermal field. All domains are defined as ’fluid’, and the initial temperature of the internal environment of the equipment is set to 293.15 K. Using the ’inflow’ boundary condition, the nozzle injection temperature is set to 293.15 K; using the ’heat source’ boundary condition, the calciner, product hopper and filter area are set as generalized sources; using the ’temperature 1’ boundary condition, the calcination furnace area is set to 400–800°C; using the ’temperature 2’ boundary condition, the temperature of the product hopper and filter area is set to 200°C.

In the simulation, the ’dilute matter transfer’ model is used to simulate the internal chemical reaction, and the simulated chemical reaction equations are shown in (11) and (12). The initial concentration of HNO_3_ and NaNO_3_ in the calcination furnace and product hopper was set as 48mol/m^3^. Using the ’inflow’ boundary condition, the concentration of C_12_H_22_O_11_ was set to 1mol/m^3^. Using the ’reaction’ boundary condition, the reaction rate *R*_i_ is defined to be related to the reaction temperature. Using the ’distribution condition’ boundary condition, the distribution coefficients of NO_2_, CO_2_ and H_2_O are set to be 1, the region is the interface between the filter inlet and the product hopper, and the distribution coefficients of the remaining substances are 0.


$$48HNO_{3}+C_{12}H_{22}O_{11}\to 48NO_{2}↑+12CO_{2}↑+35H_{2}O↑$$ (11)

$$48NaNO_{3}+C_{12}H_{22}O_{11}\to 24Na_{2}O+48NO_{2}↑+12CO_{2}↑+11H_{2}O↑$$ (12)

### 3. 3 Calculation conditions

According to the actual operation of the test device, the calculation conditions are set. Temperature is one of the important factors affecting the chemical reaction rate and product yield. In the simulation, the influence of calcination furnace temperature on chemical reaction is studied. The temperature variable is set to 400–800°C. The effects of the concentration of reactants (NaNO_3_ and HNO_3_) on the concentration of main products (NO_2_ and Na_2_O) were investigated. The concentrations of reactants (NaNO_3_ and HNO_3_) were set to 12mol/m^3^, 24mol/m^3^, 36mol/m^3^, 48mol/m^3^ and 60mol/m^3^, respectively. The boundary conditions are shown in Table 1.

**Table 1:** Boundary conditions.

Variable	Numerical value				
Calciner temperature	400°C	500°C	600°C	700°C	800°C
Concentration of reactants (NaNO_3_ and HNO_3_)	12 mol/m^3^	24 mol/m^3^	36 mol/m^3^	48 mol/m^3^	60 mol/m^3^

## 4. Numerical calculation results experimental verification

**Fig 4:**
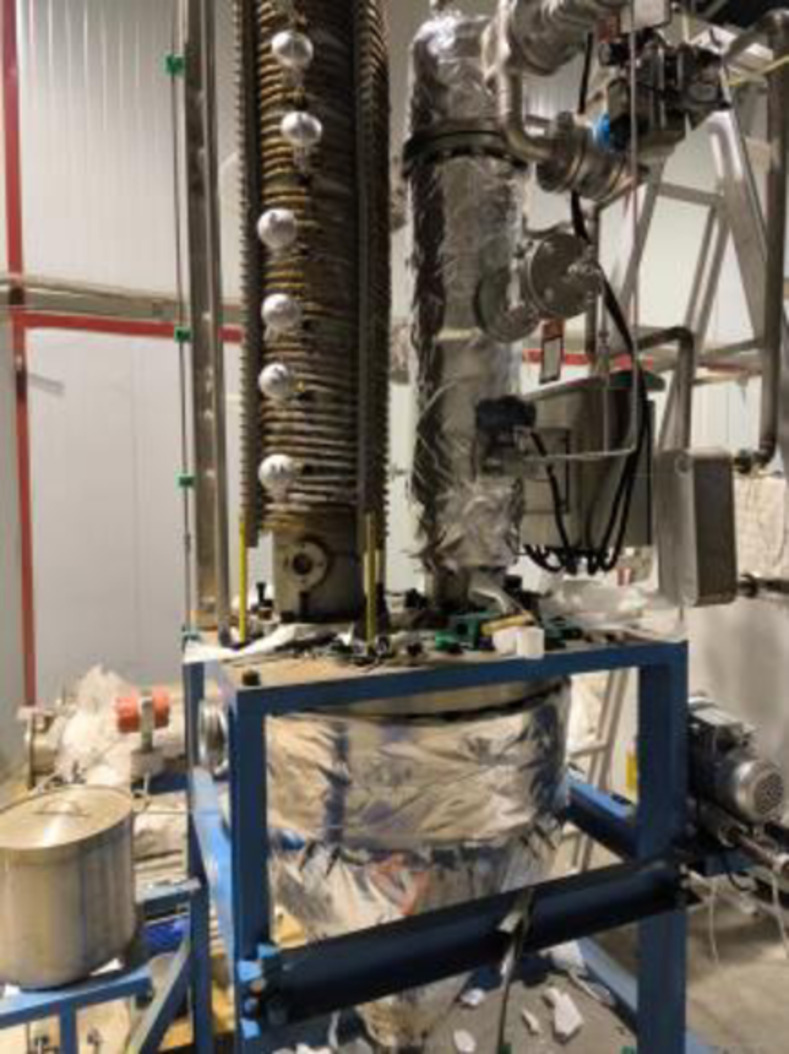
Physical diagram of high-level waste liquid spray calcination transformation treatment test device.

The simulated high-level waste liquid spray calcination transformation treatment test device is shown in Fig 4. The central control multi-function display screen can display the numerical results of heating temperature measurement. The specific value of temperature changing with time is shown in Table 2. Through experimental verification, the error between the temperature test results and the calculation results is less than 10%, indicating the accuracy of the numerical calculation results.

**Table 2:** Numerical results of heating temperature changing with time.

Time/min	Temperature/°C
0	26.67
20	170.69
40	346.36
60	483.97
80	578.29
100	640.71
120	679.61
140	705.98
160	724.23
180	735.27
200	744.49
220	750.95
240	761.01

## 5. Analysis of numerical results

### 5. 1 Analysis of flow field temperature field

The flow field and temperature field were analyzed by taking the calculation condition of ’sucrose concentration of 1mol/m^3^, NaNO_3_ and HNO_3_ concentration of 48mol/m^3^, calcination furnace temperature of 800°C as an example.

**Fig 5:**
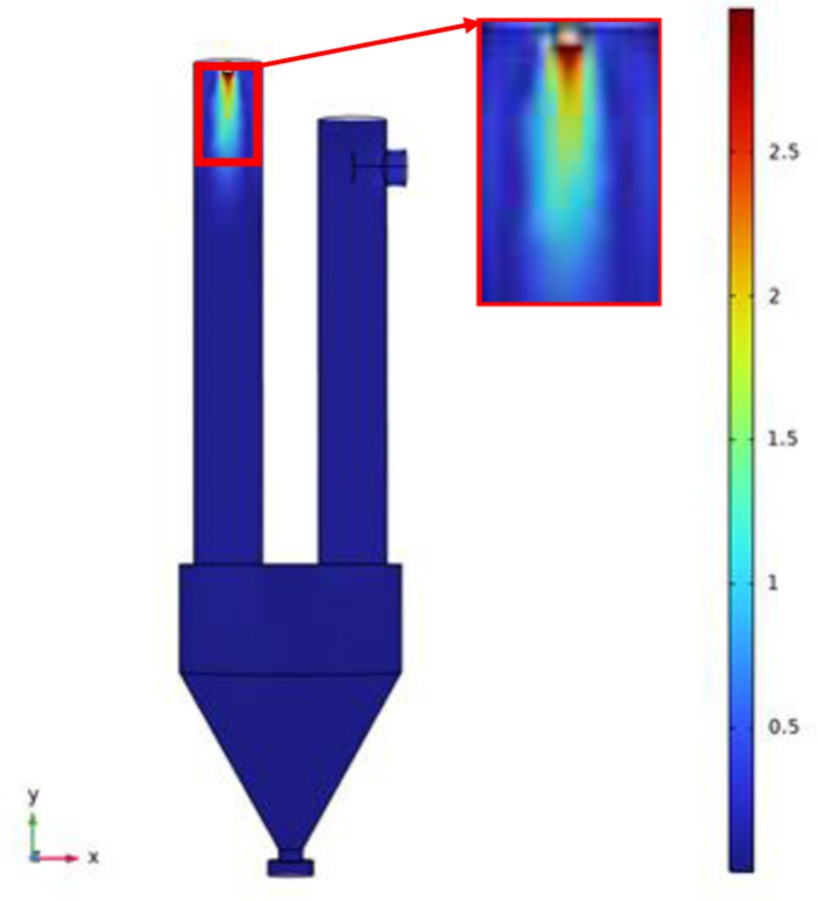
Internal streamline diagram of simulation model.

Fig 5 is the internal flow chart of the simulation model. The velocity cloud diagram clearly reflects the velocity distribution and spray shape of the liquid injected from the nozzle at the maximum speed of 3m/s. The inlet velocity is the largest, and as the injection distance increases, the velocity gradually decreases, which is consistent with the theoretical expectation.

**Fig 6:**
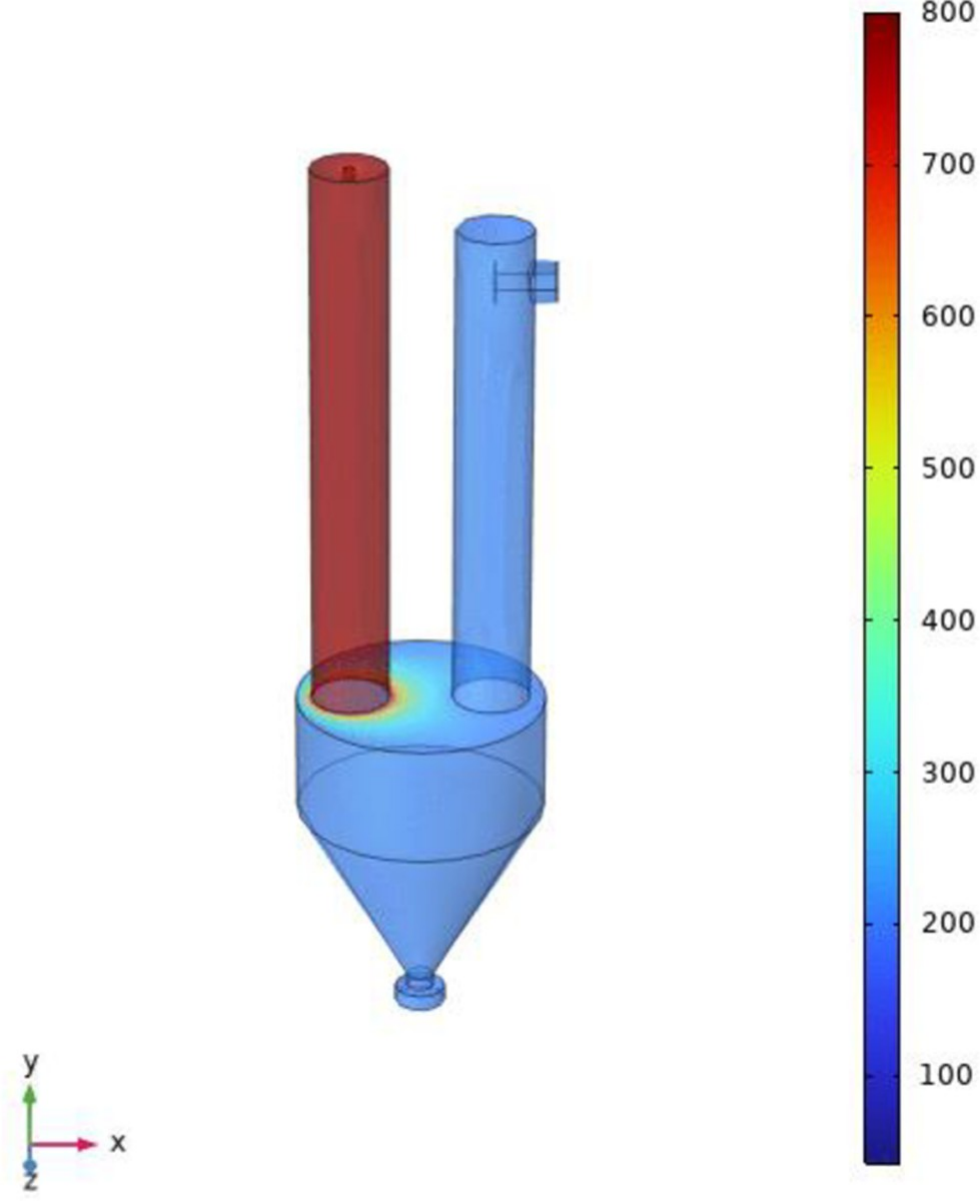
Temperature cloud diagram of simulation model.

Fig 6 is the temperature cloud diagram of the simulation model. It can be seen that the temperature of the calcination furnace area is 800°C, the temperature of the product hopper and the filter area is 200°C, and the heat transfer occurs in the contact area between the calcination furnace and the product hopper. Through the contact area cloud diagram of the calciner and the product hopper, it can be seen that as the distance between the contact surface and the heat source (calciner) increases, the temperature gradually decreases, and the heat transfer properties are consistent with the theoretical expectations, which indirectly proves the accuracy of the simulation results.

### 5. 2 Analysis of chemical reaction results

**Fig 7:**
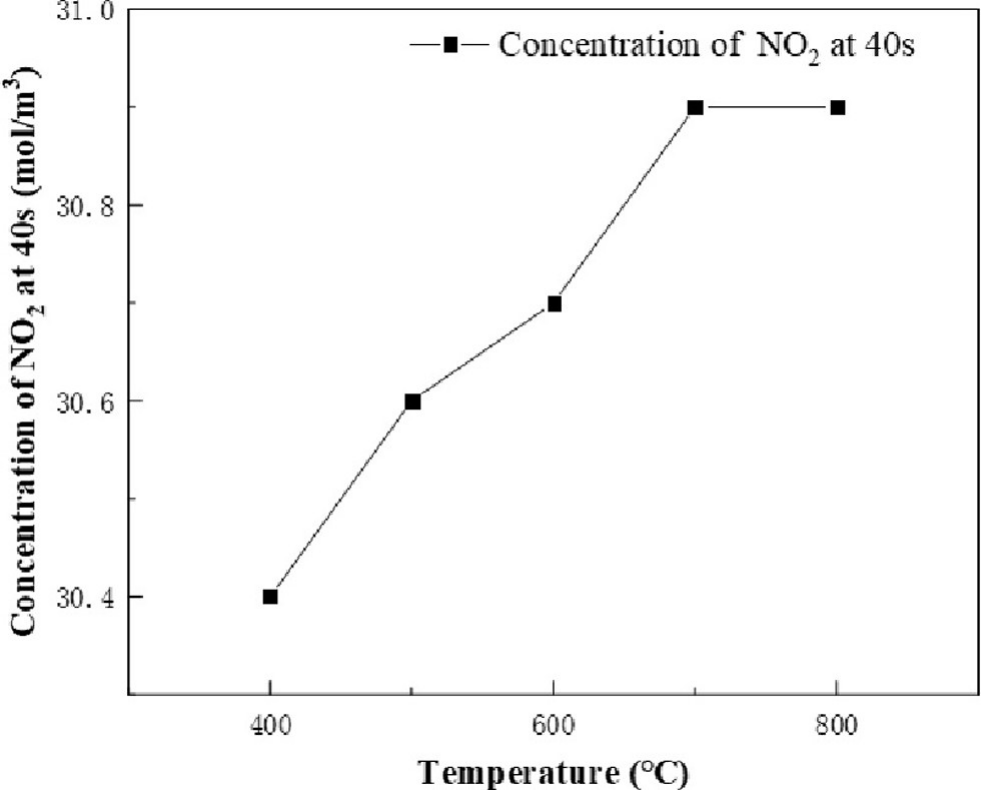
NO_2_ concentration vs. temperature at 40s.

**Fig 8:**
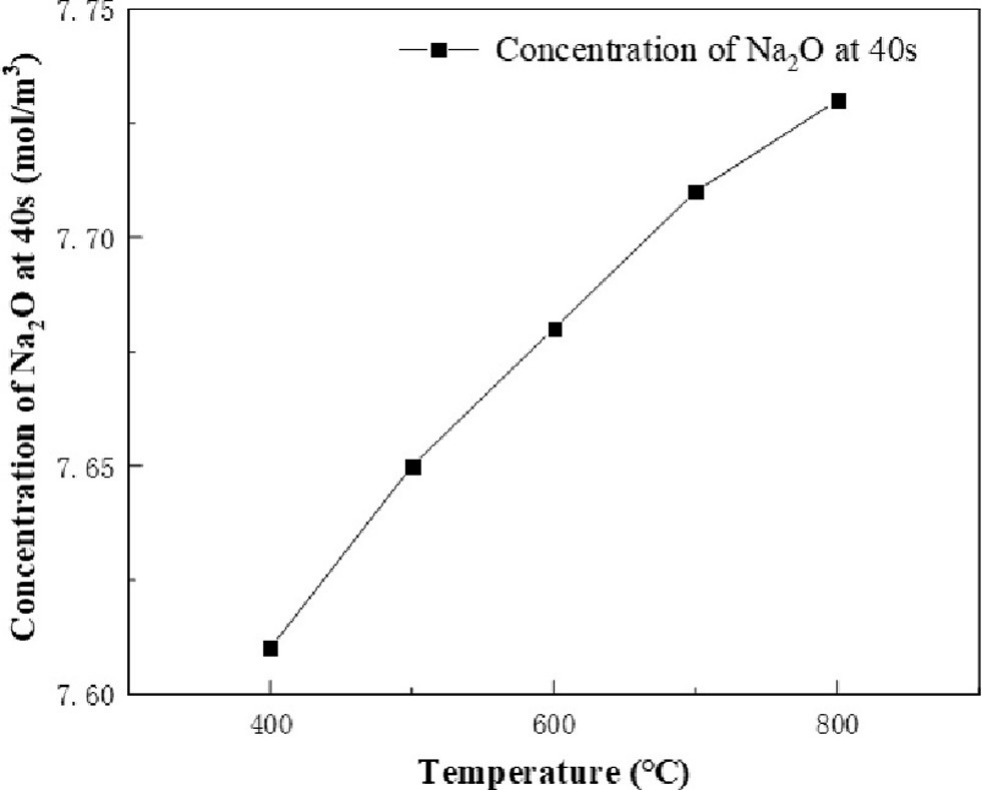
Na_2_O concentration vs. temperature at 40s.

The results of the chemical reaction inside the equipment were analyzed. According to the simulation results, it was found that the product reached the bottom of the calciner at about 40s of the chemical reaction. At this time point, the effect of calcination on the chemical reaction is over, so the product concentration at 40s is more accurate, which can truly reflect the effect of calcination furnace temperature on the chemical reaction. The calculation conditions are as follow: the concentration of sucrose is 1mol/m^3^, the concentration of NaNO_3_ and HNO_3_ is 48mol/m^3^. The calcination furnace temperature variable is set to 400–800°C, and the concentrations of NO_2_ and Na_2_O at 400°C, 500°C, 600°C, 700°C and 800°C for 40s are recorded, as shown in Figs 7 and 8. The temperature has an effect on the yield of the product. With the increase of temperature, the concentration of NO_2_ and Na_2_O increases. It can be found from the specific values that the concentration of NO_2_ increased by 0.5 mol/m^3^ and the concentration of Na_2_O increased by 0.12 mol/m^3^ at the same time when the temperature rise was also 400°C, indicating that in this chemical reaction, the effect of temperature on the yield of NO_2_ was greater than that of Na_2_O.

**Fig 9:**
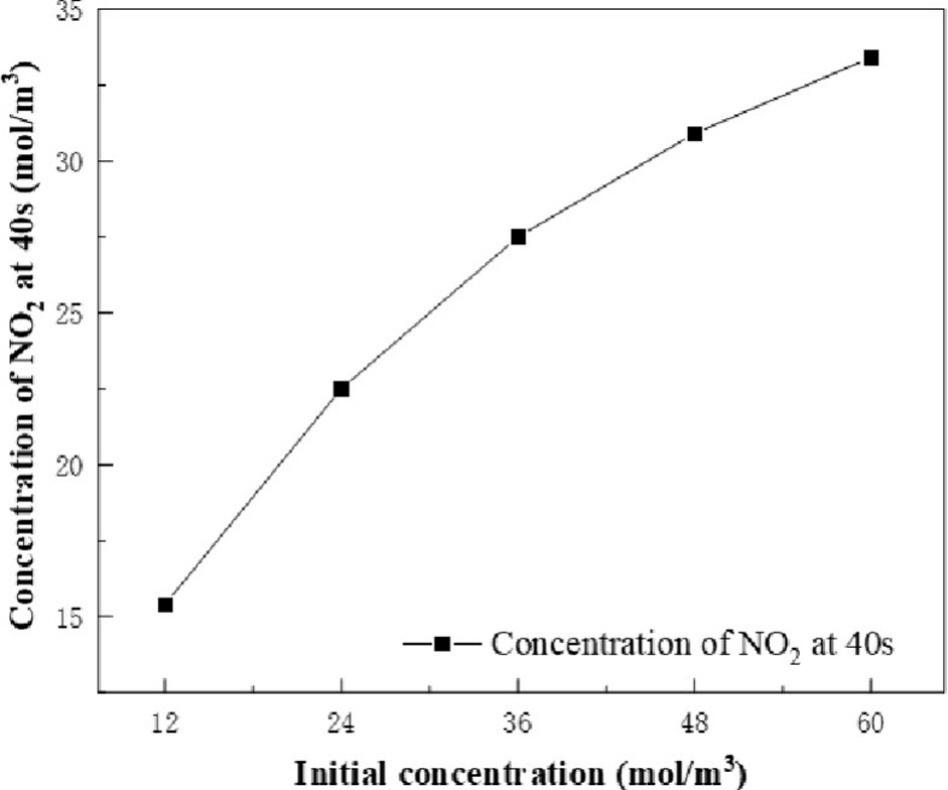
Change of NO_2_ concentration with initial reactant concentration at 40s.

**Fig 10:**
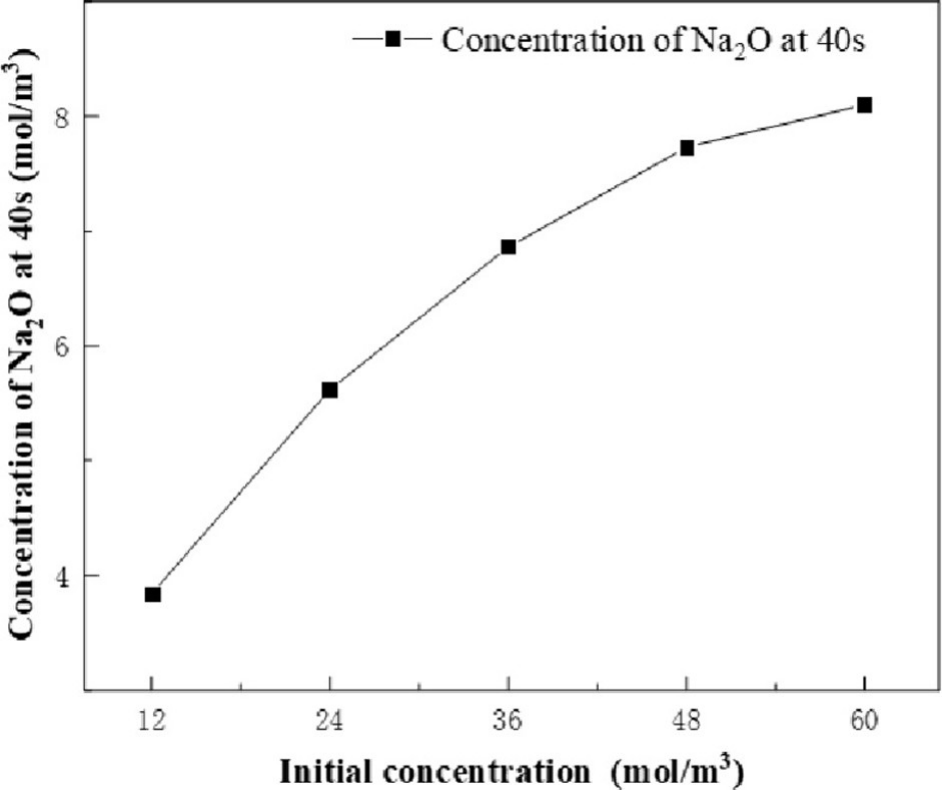
Change of Na_2_O concentration with initial reactant concentration at 40s.

The effects of the concentration of reactants (NaNO_3_ and HNO_3_) on the concentration of main products (NO_2_ and Na_2_O) were investigated by simulation. The calculation conditions are as follow: The sucrose concentration is 1 mol/m^3^, and the calcination furnace temperature is set to 800°C. The concentration of reactants was set as a variable, and the effects of NaNO_3_ and HNO_3_ concentrations of 12 mol/m^3^, 24 mol/m^3^, 36 mol/m^3^, 48 mol/m^3^ and 60 mol/m^3^ on the concentration of NO_2_ and Na_2_O at 40s were investigated. The simulation results are shown in Figs 9 and 10. The concentration of reactants (NaNO_3_ and HNO_3_) has an effect on the concentration of the product. As the concentration of reactants (NaNO_3_ and HNO_3_) increases, the concentration of NO_2_ and Na_2_O increases. Taking NO_2_ as an example, it can be found that the concentration of reactants (NaNO_3_ and HNO_3_) increased from 12 mol/m^3^ to 24 mol/m^3^, and the concentration of NO_2_ increased by 7.1 mol/m^3^. The concentration of reactants (NaNO_3_ and HNO_3_) increased from 48 mol/m^3^ to 60 mol/m^3^, and the concentration of NO_2_ increased by 0.5 mol/m^3^. Similarly, the product Na_2_O can also draw the same conclusion. Therefore, the higher the concentration of reactants (NaNO_3_ and HNO_3_), the lower the rate of increase in the concentration of the main products (NO_2_ and Na_2_O).

**Fig 11:**
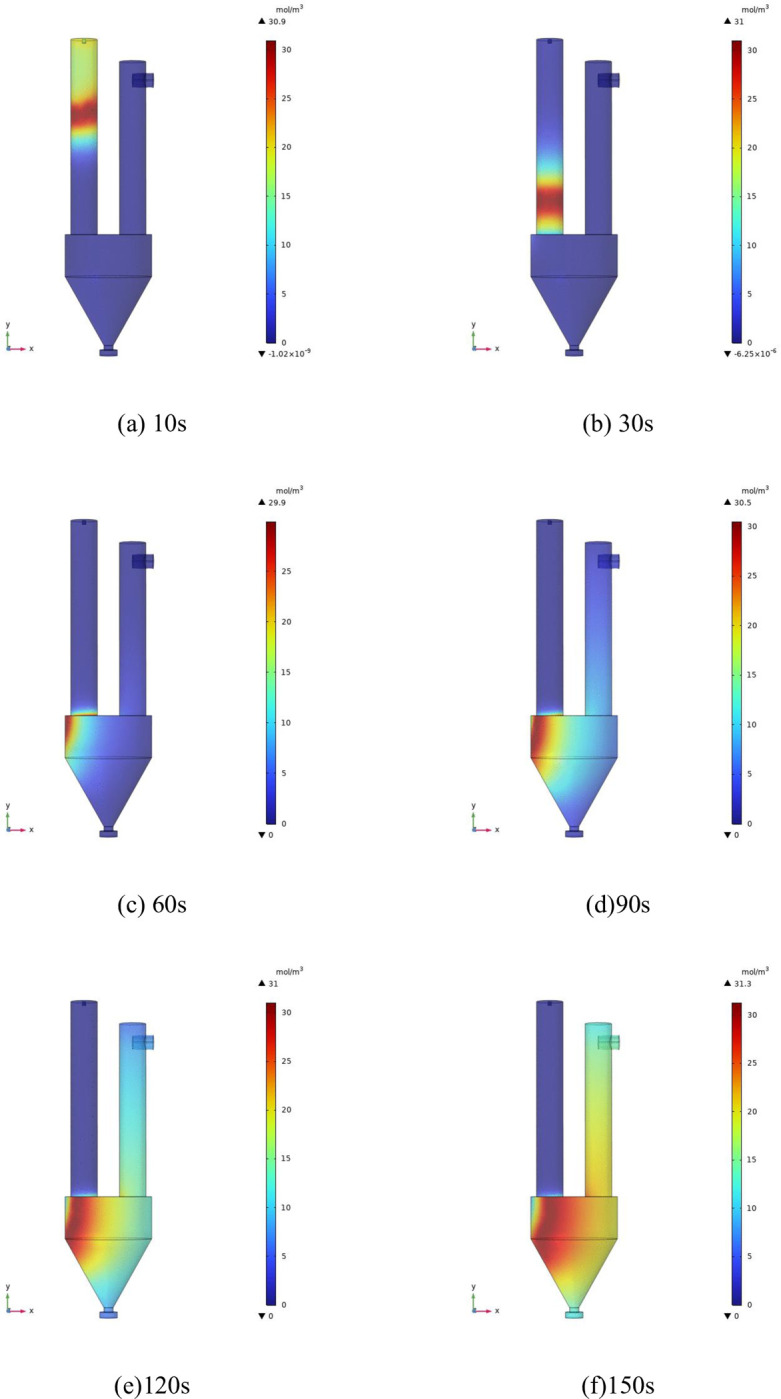
The cloud chart of NO_2_ concentration changing with time.

The calculation condition is ’sucrose concentration is 1mol/m^3^, NaNO_3_ and HNO_3_ concentration is 48mol/m^3^, calcining furnace temperature is 800°C. Taking the cloud map of NO_2_ concentration changing with time (as shown in Fig 11) as an example, the chemical reaction process in internal spray calcination is shown. Through the cloud map, it can be seen that with the change of time, NO_2_ is generated in the calciner, flows down the calciner into the product hopper, and then diffuses into the filter. It is consistent with the streamline diagram and the actual experiment. The accuracy of the experimental results is proved.

## 6. Conclusion and foresight

In view of the independent research and development of a simulated high-level liquid waste spray calcination and transformation treatment test device, a multi-physical field coupling calculation model for spray calcination was established. The specific conclusions and prospects are as follows:

1. In this chemical reaction, temperature is an important factor. Temperature has an effect on the yield of the product. With the increase of temperature, the concentration of NO_2_ and Na_2_O increases continuously, and the effect of temperature on the yield of NO_2_ is greater than that of Na_2_O.
2. In this chemical reaction, the concentration of reactants (NaNO_3_ and HNO_3_) has an effect on the concentration of the product. With the increase of the concentration of reactants (NaNO_3_ and HNO_3_), the concentration of NO_2_ and Na_2_O increases. At the same time, the higher the concentration of reactants (NaNO_3_ and HNO_3_), the lower the rate of increase in the concentration of main products (NO_2_ and Na_2_O).
3. The current calculation model lacks the tracking of spray particles and cannot track the trajectory of spray particles. In addition, the spray cone angle is currently achieved through the simplification of the nozzle structure, which is not real enough and can be further improved in the model in the future.

## Supporting information

S1 Raw data The numerical calculation of the original data contains all the data points that make up the statistical chart. (DOCX)

## References

[1] LONG Lin, TIAN Yingnan, YOU Wei, et al. The safety analysis of high-level liquid waste store [J]. Nuclear Safety, 2016, 15(01): 23–29+37. doi: 10.16432/j.cnki.1672-5360.2016.01.004

[2] ZHONG Yun, FU Yunshan, GAO Panwei, et al. Retrieval methods and techniques for tank storage HLLW [J]. Guangdong Chemical Industry, 2015, 42(5): 172–175.

[3] ZHOU Zizheng, LIU Yingzi, WANG Zhiping, et al. Analysis of factors affecting noble metal lons deposition during glass melting [J]. Guangdong Chemical Industry, 2023, 50(06): 167–169.

[4] ZHANG Hua, LI Baojun, LI Yang, et al. Study on influence factors of calcined products from simulated HLLW based on gaussian kriging agent model [J], Journal of Nuclear and Radiochemistry, 2001, 21(4): 264–267. doi: 10.7538/hhx.2023.45.01.0076

[5] LUO Shanggeng. Radioactive waste treatment and disposal [M]. Beijing: China Environmental Press, 2007.

[6] LUO Shanggeng. Separation and transmutation of high-level radioactive waste [J]. Radiation Protection, 1996(1): 72–75.

[7] LIU Lijun, ZHANG Shengdong, et al. Analysis of technical development of vitrificating radioactive waste in cold crucible induction melter [J]. Atomic Energy Science and Technology, 2015, 49(04): 589–596. DOI: 10.7538/yzk.2015.49.04.0589.

[8] LIANG Xuan, JIA Yunquan, LI Zhongdi, et al. Research actuality and development prospect of two-steps cold-crucible vitrification technology [J]. Modern Chemical Research, 2023(08): 14–16. doi: 10.20087/j.cnki.1672-8114.2023.08.005

[9] ZHANG Anqi, WANG Zexue, LONG Haoqi, et al. Fault diagnosis and improvement of calciner in high-level liquid waste water cold crucible system based on analytic hierarchy process [J]. Guangdong Chemical Industry, 2023, 50(03): 110–112.

[10] WANG Zexue, LI Baojun, LI Yusong, et al. Design of adaptive pressure regulating system for glass solidification of HLLW by cold crucible [J]. Atomic Energy Science and Technology, 2022, 56(12): 2607–2615. doi: 10.7538/yzk.2021youxian.0523

[11] LI Jiangbo, ZHANG Shengdong, et al. Present situation of research on calcine technology of high-level liquid waste [J]. Journal of Nuclear and Radiochemistry, 2014, (201): 1–5. doi: 10.7538/hhx.2014.36.S0.0001

[12] Bjorklund W. J.. Fluidized bed calcination experience with simulated commercial high-level nuclear waste [M]. Richland: Battelle Pacific Northwest Laboratories, 1976.

[13] Nan LIU. Numerical Simulation and Experimental Study on Coupled Flow in Spray Drying [D]. Dalian University of Technology, 2021.

[14] WU Zhonghua LIU Xiangdong. Simulation of Spraying Drying of a Solution Atomized in a Pulsating Flow [J]. Drying Technology, 2002, 20(6): 1101–1121. doi: 10.7538/hhx.2021.YX.2020046

[15] George O. A., Xiao J., Rodrigo C. S., et al. Detailed numerical analysis of evaporation of a micrometer water droplet suspended on a glass filament [J]. Chemical Engineering Science, 2017, 165: 33–47.

[16] Volkov R. S., Strizhak P. A.. Planar laser-induced fluorescence diagnostics of water droplets heating and evaporation at high-temperature [J]. Applied Thermal Engineering, 2017, 127: 141–156.

[17] Penghua GUO, Tiantian Li, Peiwen Li, et al. Study on a novel spray-evaporation multi-effect distillation de salination system [J]. Desalination, 2020, 473:114195.

[18] MA Shuangchen, CHAI Jin, CHEN Jianing, et al. Numerical simulation of bypass evaporation system treating FGD wastewater using high temperature flue gas [J]. International journal of sports medicine, 2020, 41(6): 751–763. 30095048 10.1080/09593330.2018.1509892

