# Supplementary material for: Multi-physical field simulation calculation and analysis of simulated high-level waste liquid spray calcination
Source: PLoS One. 2024 Aug 9;19(8):e0308145. doi: 10.1371/journal.pone.0308145 (PMC11315273; doi:10.1371/journal.pone.0308145)
Supplement: S1 Raw data — (DOCX) [file pone.0308145.s001.docx]

Dear Editors and Reviewers:

The original data used to construct the graph and its analysis are as follows:

| 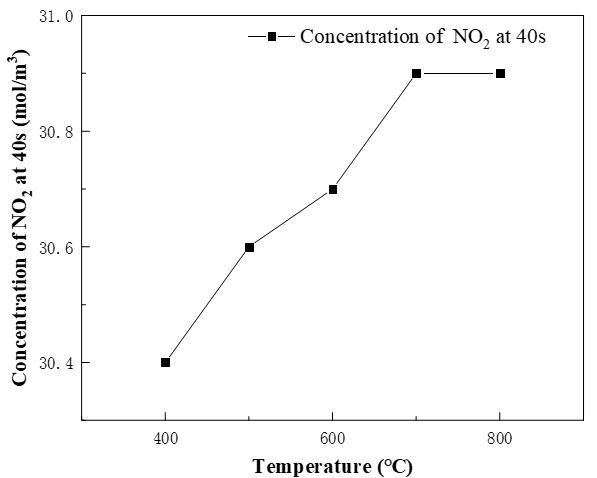 | 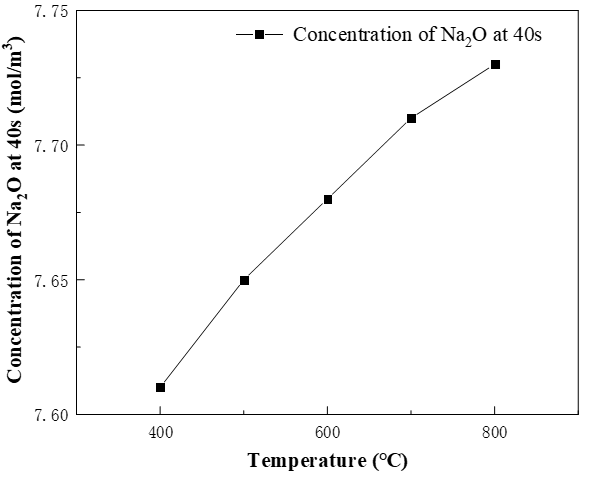 |
| --- | --- |
| Fig. 1 NO_2_ concentration vs. temperature at 40s | Fig. 2 Na_2_O concentration vs. temperature at 40s |

Table1 Figure 1 and Figure 2 raw data

| Temperature(℃) | The concentration of Na_2_O at 40s(mol/m^3^) | The concentration of NO_2_ at 40s(mol/m^3^) |
| --- | --- | --- |
| 400 | 7.61 | 30.4 |
| 500 | 7.65 | 30.6 |
| 600 | 7.68 | 30.7 |
| 700 | 7.71 | 30.9 |
| 800 | 7.73 | 30.9 |

The calcination furnace temperature variable is set to 400-800 °C, and the concentrations of NO_2_ and Na_2_O at 400 °C, 500 °C, 600 °C, 700 °C and 800 °C for 40s are recorded, as shown in Fig.1 and Fig.2. The temperature has an effect on the yield of the product. With the increase of temperature, the concentration of NO_2_ and Na_2_O increases. It can be found from the specific values that the concentration of NO_2_ increased by 0.5 mol/m^3^ and the concentration of Na_2_O increased by 0.12 mol/m^3^ at the same time when the temperature rise was also 400 °C, indicating that in this chemical reaction, the effect of temperature on the yield of NO_2_ was greater than that of Na_2_O.

| 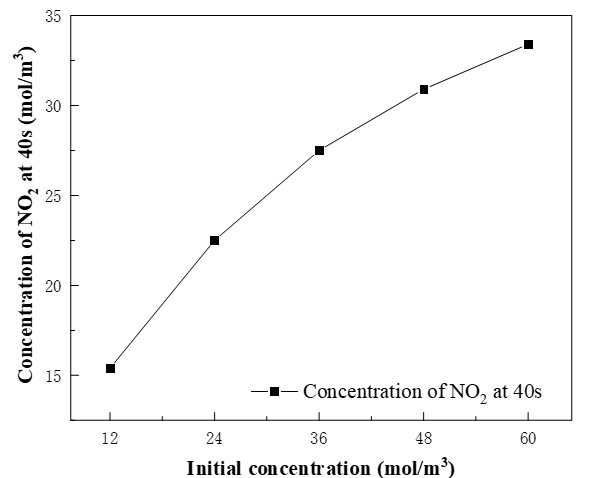 | 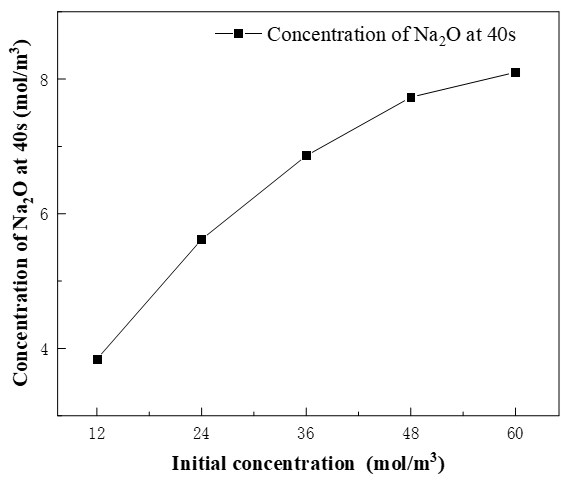 |
| --- | --- |
| Fig. 3 Change of NO_2_ concentration with initial reactant concentration at 40s | Fig. 4 Change of Na_2_O concentration with initial reactant concentration at 40s |

Table2 Figure 3 and Figure 4 raw data

| The initial concentration of NaNO_3_ and HNO_3_(mol/m^3^) | The concentration of Na_2_O at 40s(mol/m^3^) | The concentration of NO_2_ at 40s(mol/m^3^) |
| --- | --- | --- |
| 12 | 3.84 | 15.4 |
| 24 | 5.62 | 22.5 |
| 36 | 6.86 | 27.5 |
| 48 | 7.73 | 30.9 |
| 60 | 8.1 | 33.4 |

The concentration of reactants was set as a variable, and the effects of NaNO_3_ and HNO_3_ concentrations of 12 mol/m^3^,24 mol/m^3^,36 mol/m^3^,48 mol/m^3^ and 60 mol/m^3^ on the concentration of NO_2_ and Na_2_O at 40s were investigated. The simulation results are shown in Fig.3 and Fig.4. The concentration of reactants (NaNO_3_ and HNO_3_) has an effect on the concentration of the product. As the concentration of reactants (NaNO_3_ and HNO_3_) increases, the concentration of NO_2_ and Na_2_O increases. Taking NO_2_ as an example, it can be found that the concentration of reactants (NaNO_3_ and HNO_3_) increased from 12 mol/m^3^ to 24 mol/m^3^, and the concentration of NO_2_ increased by 7.1 mol/m^3^. The concentration of reactants (NaNO_3_ and HNO_3_) increased from 48 mol/m^3^ to 60 mol/m^3^, and the concentration of NO_2_ increased by 0.5 mol/m^3^. Similarly, the product Na_2_O can also draw the same conclusion. Therefore, the higher the concentration of reactants (NaNO_3_ and HNO_3_), the lower the rate of increase in the concentration of the main products (NO_2_ and Na_2_O).
